# Supplementary material for: Single-cell multiomics of the human retina reveals hierarchical transcription factor collaboration in mediating cell type-specific effects of genetic variants on gene regulation
Source: Genome Biol. 2023 Nov 27;24:269. doi: 10.1186/s13059-023-03111-8 (PMC10680294; doi:10.1186/s13059-023-03111-8)
Supplement: Supplementary file 1 — Additional file 1: Fig. S1. The OCRs identified from snATAC-seq. Fig. S2. Identification of sc-eQTL. Fig. S3. Identification of sc-caQTL. Fig. S4. Identification of sc-caQTL in down-sampled cells. Fig. S5. Cell type enrichment and fine-mapping of GWAS loci. Fig. S6. An example of the fine-mapped GWAS candidate variants with the retinal bulk eQTL signal of its target gene colocalized with GWAS signal. Supplementary Note. Acronyms list. [file 13059_2023_3111_MOESM1_ESM.pdf]

Supplementary Information

Single-cell multiomics of the human retina reveals hierarchical transcription factor collaboration in mediating cell type-specific effects of genetic variants on gene regulation

Jun Wang[1,2,8], Xuesen Cheng[1,2,8], Qingnan Liang[1,2,3], Leah A. Owen[4], Jiaxiong Lu[1,2,3], Yiqiao Zheng[5], Meng Wang[1,2], Shiming Chen[5,6], Margaret M. DeAngelis[7], Yumei Li[1,2], Rui Chen[1,2,*]

[1]Human Genome Sequencing Center, Baylor College of Medicine, Houston, TX, USA
[2]Department of Molecular and Human Genetics, Baylor College of Medicine, Houston, TX, USA
[3]Verna and Marrs McLean Department of Biochemistry and Molecular Biology, Baylor College of Medicine, Houston, TX, USA
[4]John A. Moran Eye Center, Department of Ophthalmology and Visual Sciences, University of Utah, Salt Lake City, UT, USA
[5]Department of Ophthalmology and Visual Sciences, Washington University in St Louis, Saint Louis, MO, USA
[6]Department of Developmental Biology, Washington University in St Louis, Saint Louis, MO, USA
[7]Department of Ophthalmology, University at Buffalo the State University of New York, Buffalo, NY, USA

[8]These authors contributed equally
[*]Correspondence to be addressed to Rui Chen: ruichen@bcm.edu

Supplementary Figures

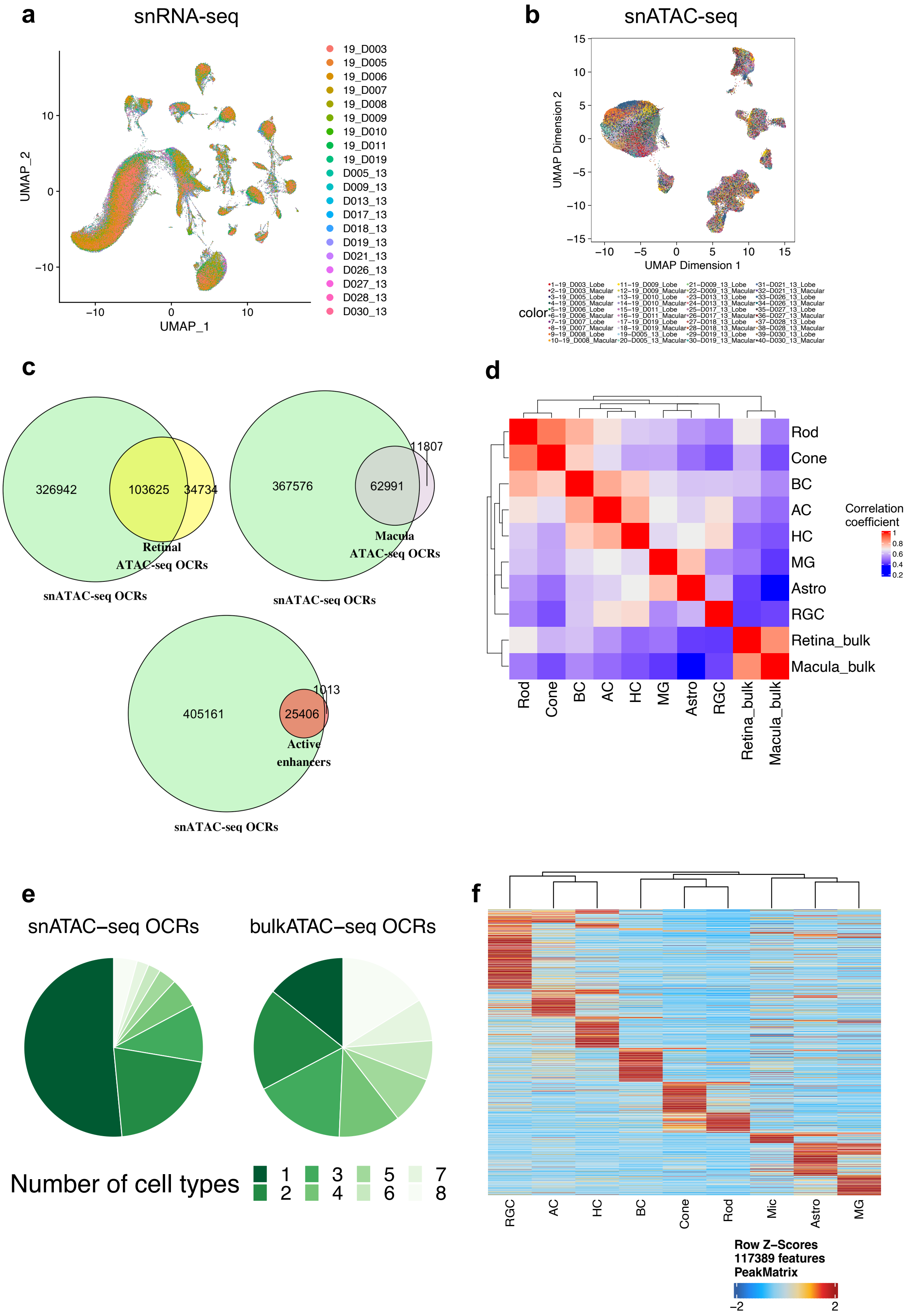
a
snRNA-seq
UMAP_2
UMAP_1
19_D003
19_D005
19_D006
19_D007
19_D008
19_D009
19_D010
19_D011
19_D019
D005_13
D009_13
D013_13
D017_13
D018_13
D019_13
D021_13
D026_13
D027_13
D028_13
D030_13
b
snATAC-seq
UMAP Dimension 2
UMAP Dimension 1
color
1–19_D003_Lobe
2–19_D003_Macular
3–19_D005_Lobe
4–19_D005_Macular
5–19_D006_Lobe
6–19_D006_Macular
7–19_D007_Lobe
8–19_D007_Macular
9–19_D008_Lobe
10–19_D008_Macular
11–19_D009_Lobe
12–19_D009_Macular
13–19_D010_Lobe
14–19_D010_Macular
15–19_D011_Lobe
16–19_D011_Macular
17–19_D019_Lobe
18–19_D019_Macular
19–D005_13_Lobe
20–D005_13_Macular
21–D009_13_Lobe
22–D009_13_Macular
23–D013_13_Lobe
24–D013_13_Macular
25–D017_13_Lobe
26–D017_13_Macular
27–D018_13_Lobe
28–D018_13_Macular
29–D019_13_Lobe
30–D019_13_Macular
31–D021_13_Lobe
32–D021_13_Macular
33–D026_13_Lobe
34–D026_13_Macular
35–D027_13_Lobe
36–D027_13_Macular
37–D028_13_Lobe
38–D028_13_Macular
39–D030_13_Lobe
40–D030_13_Macular
c
326942
103625
34734
Retinal ATAC-seq OCRs
snATAC-seq OCRs
367576
62991
11807
Macula ATAC-seq OCRs
snATAC-seq OCRs
405161
25406
1013
Active enhancers
snATAC-seq OCRs
d
Rod
Cone
BC
AC
HC
MG
Astro
RGC
Retina_bulk
Macula_bulk
Correlation coefficient
1
0.8
0.6
0.4
0.2
e
snATAC–seq OCRs
bulkATAC–seq OCRs
Number of cell types
1 2 3 4 5 6 7 8
f
RGC
AC
HC
BC
Cone
Rod
Mic
Astro
MG
Row Z-Scores
117389 features
PeakMatrix
–2
2

Fig. S1: The OCRs identified from snATAC-seq.

a, Uniform Manifold Approximation and Projection (UMAP) of cells from snRNA-seq. Each dot corresponds to a cell, colored by the sample/batch source of the cell. The cells were clustered into major retinal cell types. n = 192,792 cells. **b,** UMAP of cells from snATAC-seq. Each dot corresponds to a cell, colored by the sample/batch source of the cell. The cells were clustered into major retinal cell types. n = 245,940 cells. **c,** Venn diagram showing the number of OCRs identified by snATAC-seq and bulk ATAC-seq, as well as active enhancers identified from bulk ATAC-seq and bulk ChIP-seq. **d,** Heatmap showing the Pearson correlation of chromatin accessibility across retinal cell types and bulk retina and macula tissues. n=467806 OCRs. **e,** Pie chart showing the proportions of snATAC-seq OCRs and bulkATAC-seq OCRs, colored by the number of cell types where the OCRs were identified. 51.5% of the OCRs from snATAC-seq are unique to one cell type while 14.3% of the OCRs from bulk ATAC-seq are unique to one cell type. **f,** Heatmap showing chromatin accessibility of the differential accessible regions (DARs) across retinal cell types. n = 117389 OCRs.

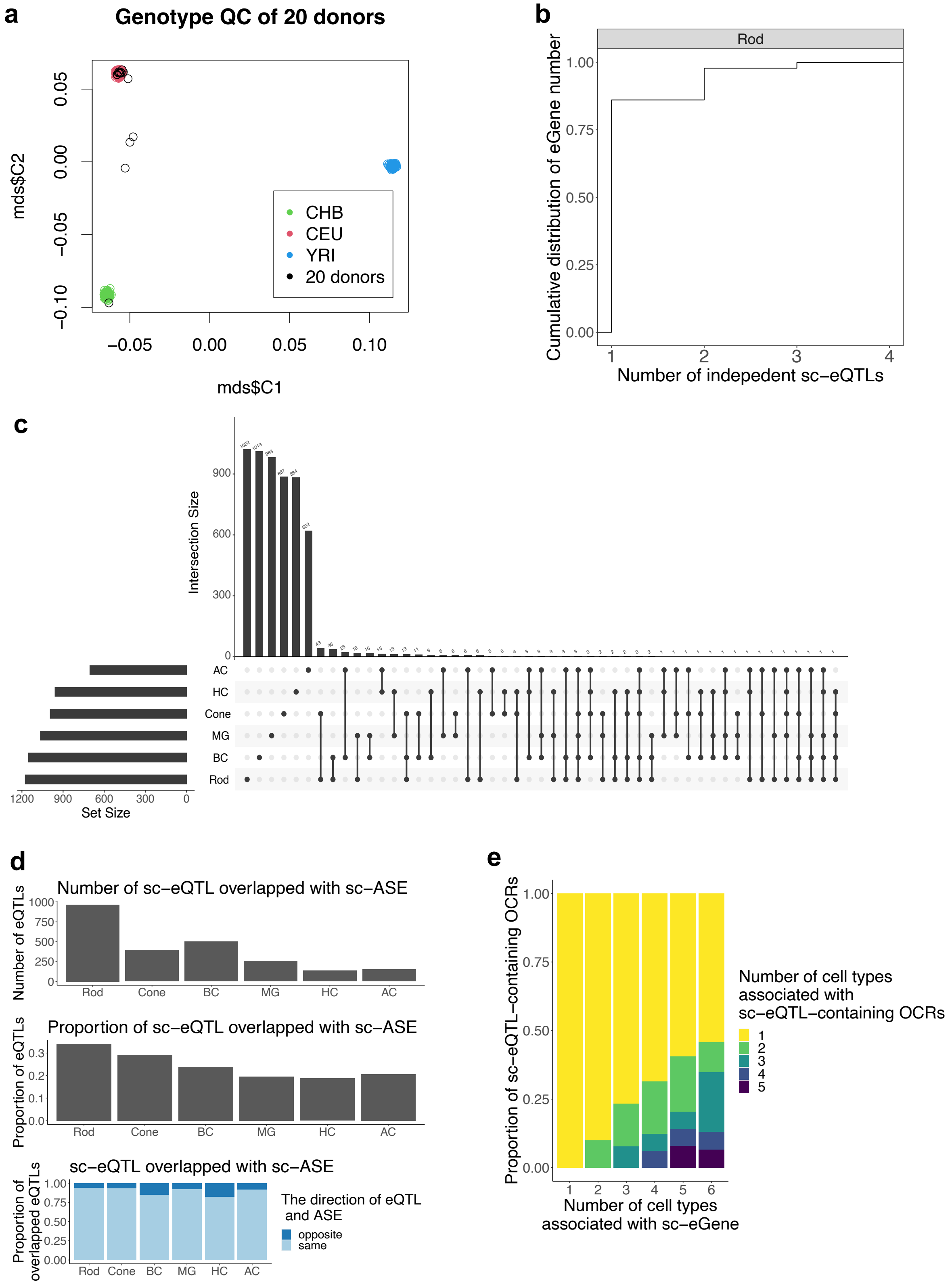
a
Genotype QC of 20 donors
mds$C2
mds$C1
0.05
0.00
−0.05
−0.10
−0.05
0.00
0.05
0.10
CHB
CEU
YRI
20 donors
b
Rod
Cumulative distribution of eGene number
1.00
0.75
0.50
0.25
0.00
1
2
3
4
Number of indepedent sc−eQTLs
c
Intersection Size
900
600
300
0
AC
HC
Cone
MG
BC
Rod
1200
900
600
300
0
Set Size
d
Number of sc−eQTL overlapped with sc−ASE
Number of eQTLs
1000
750
500
250
0
Rod
Cone
BC
MG
HC
AC
Proportion of sc−eQTL overlapped with sc−ASE
Proportion of eQTLs
0.3
0.2
0.1
0.0
Rod
Cone
BC
MG
HC
AC
sc−eQTL overlapped with sc−ASE
Proportion of overlapped eQTLs
1.00
0.75
0.50
0.25
0.00
Rod
Cone
BC
MG
HC
AC
The direction of eQTL and ASE
opposite
same
e
Proportion of sc−eQTL−containing OCRs
1.00
0.75
0.50
0.25
0.00
1
2
3
4
5
6
Number of cell types associated with sc−eGene
Number of cell types associated with sc−eQTL−containing OCRs
1
2
3
4
5

Fig. S2: Identification of sc-eQTL.

a, Scatter plot showing the 1st component (X axis) and 2nd component (Y axis) of the MDS analysis of the genotype of the 20 donors and 316 individuals from the Hapmap populations. Each dot corresponds to an individual, colored by the ethnicity of the individual. The green color corresponds to the Chinese Han population, the red color corresponds to the European population, the blue color corresponds to the African population. The 20 donor samples are colored in black. **b**, The cumulative distribution plot of sc-eGene number showing the distribution of the number of independent sc-eQTLs per sc-eGene in Rod. n = 1011 sc-eQTLs. **c,** Upset plot showing the number of sc-eQTLs across retinal cell types. **d,** Bar plot showing the number and proportion of sc-eQTLs that are overlapped with sc-ASEs across retinal cell types, based on the sc-eQTLs that were tested for sc-ASEs (top and middle). Bar plot showing the proportion of the overlapped variants with the same or oppositive effect direction between sc-eQTLs and sc-ASEs across retinal cell types (bottom). **e,** Bar plot showing the proportion of the eQTL-containing OCRs associated with eGenes that were significant in one or more cell types, colored by the number of cell types where the OCRs contain significant sc-eQTLs. The OCRs harboring sc-eQTLs are different across cell types for the same sc-eGenes common in multiple cell types. n = 3447 OCRs for sc-eGenes associated with 1 cell type, n = 1368 for 2 cell types, n = 416 for 3 cell types, n = 162 for 4 cell types, n = 64 for 5 cell types, n = 46 for 6 cell types.

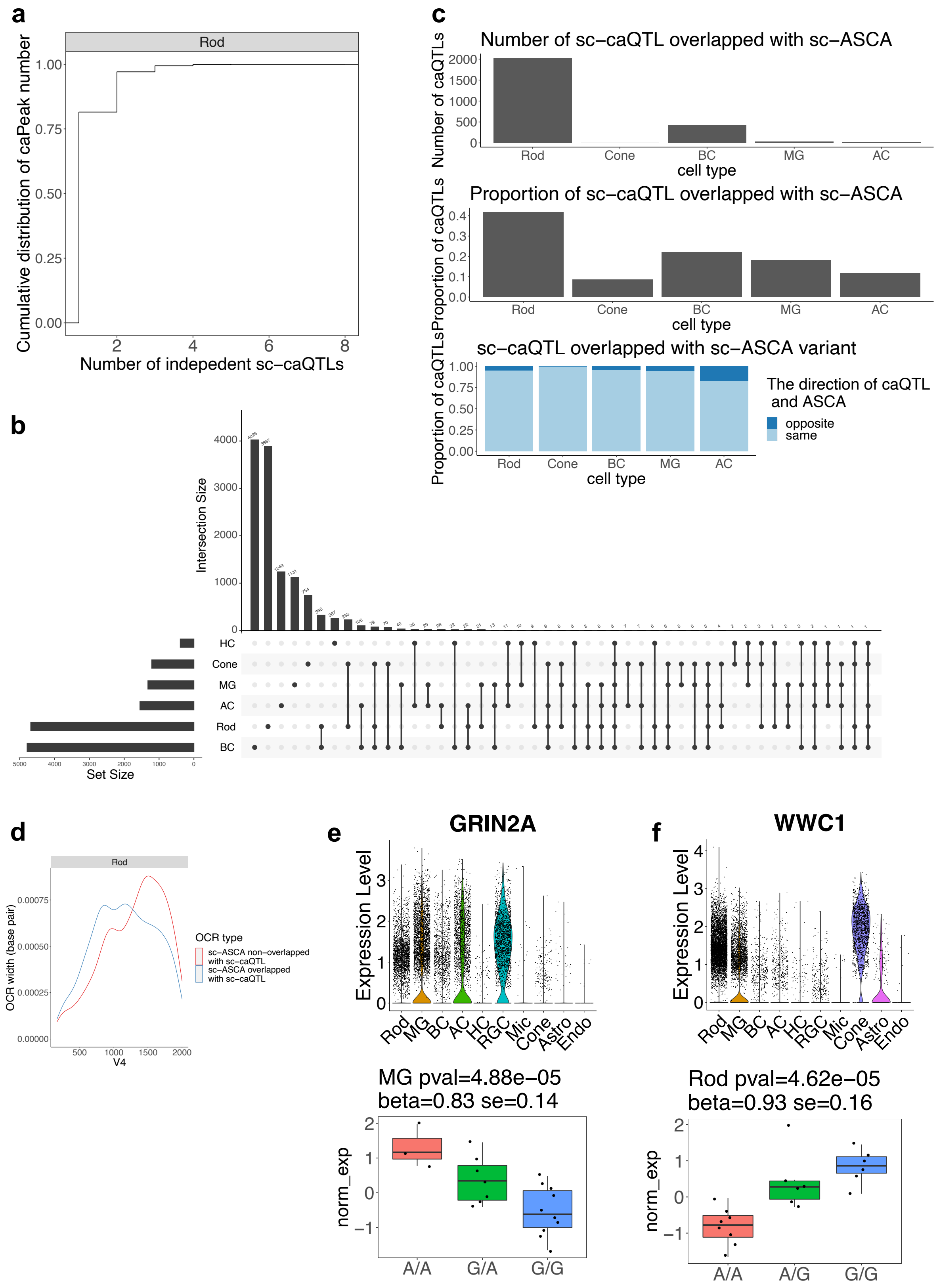
a
Rod
Cumulative distribution of caPeak number
Number of indepedent sc−caQTLs
b
Intersection Size
Set Size
HC
Cone
MG
AC
Rod
BC
c
Number of sc−caQTL overlapped with sc−ASCA
Number of caQTLs
cell type
Proportion of sc−caQTL overlapped with sc−ASCA
Proportion of caQTLs
sc−caQTL overlapped with sc−ASCA variant
The direction of caQTL and ASCA
opposite
same
d
Rod
OCR width (base pair)
V4
OCR type
sc−ASCA non−overlapped with sc−caQTL
sc−ASCA overlapped with sc−caQTL
e
GRIN2A
Expression Level
Rod MG BC AC HC RGC Mic Cone Astro Endo
MG pval=4.88e−05
beta=0.83 se=0.14
norm_exp
A/A G/A G/G
f
WWC1
Expression Level
Rod MG BC AC HC RGC Mic Cone Astro Endo
Rod pval=4.62e−05
beta=0.93 se=0.16
norm_exp
A/A A/G G/G

Fig. S3: Identification of sc-caQTL.

a, The cumulative distribution plot of sc-caPeak number showing the distribution of the number of independent sc-caQTLs per sc-caPeak in Rod. n = 3833 sc-caQTLs. **b,** Upset plot showing the number of sc-caQTLs across cell types. **c,** Bar plot showing the number and proportion of sc-caQTLs that are overlapped with sc-ASCAs across retinal cell types, based on the sc-caQTLs that were tested for sc-ASCAs (top and middle). Bar plot showing the proportion of the overlapped variants with the same or oppositive effect direction between sc-caQTLs and sc-ASCAs across retinal cell types (bottom). **d,** Density plot showing the distribution of the width of the OCRs harboring sc-ASCA in Rod, colored by whether the sc-ASCA overlapped with sc-caQTL. To avoid the confounding effect from the potential mis-calling of the wider OCRs, we only considered the OCRs with width $\leq 2kb$. The OCRs that contain sc-ASCA not overlapped with sc-caQTL are significantly wider than the OCRs that contain sc-ASCA overlapped with sc-caQTL in Rod. One-sided Wilcoxon ranking sum test, $p = 7.7 \times 10^{-13}$. n = 1024 sc-ASCAs that are not overlapped with sc-caQTLs. n = 1086 sc-ASCAs that are overlapped with sc-caQTLs. **e,** Violin plot showing the gene expression level of *GRIN2A* across retinal cell types. Box plot showing the gene expression level of *GRIN2A* in 20 individuals with different genotypes of rs12447029 in MG. **f**, Violin plot showing the gene expression level of *WWC1* across retinal cell types. Box plot showing the gene expression level of *WWC1* in 20 individuals with different genotypes of rs6859300 in Rod.

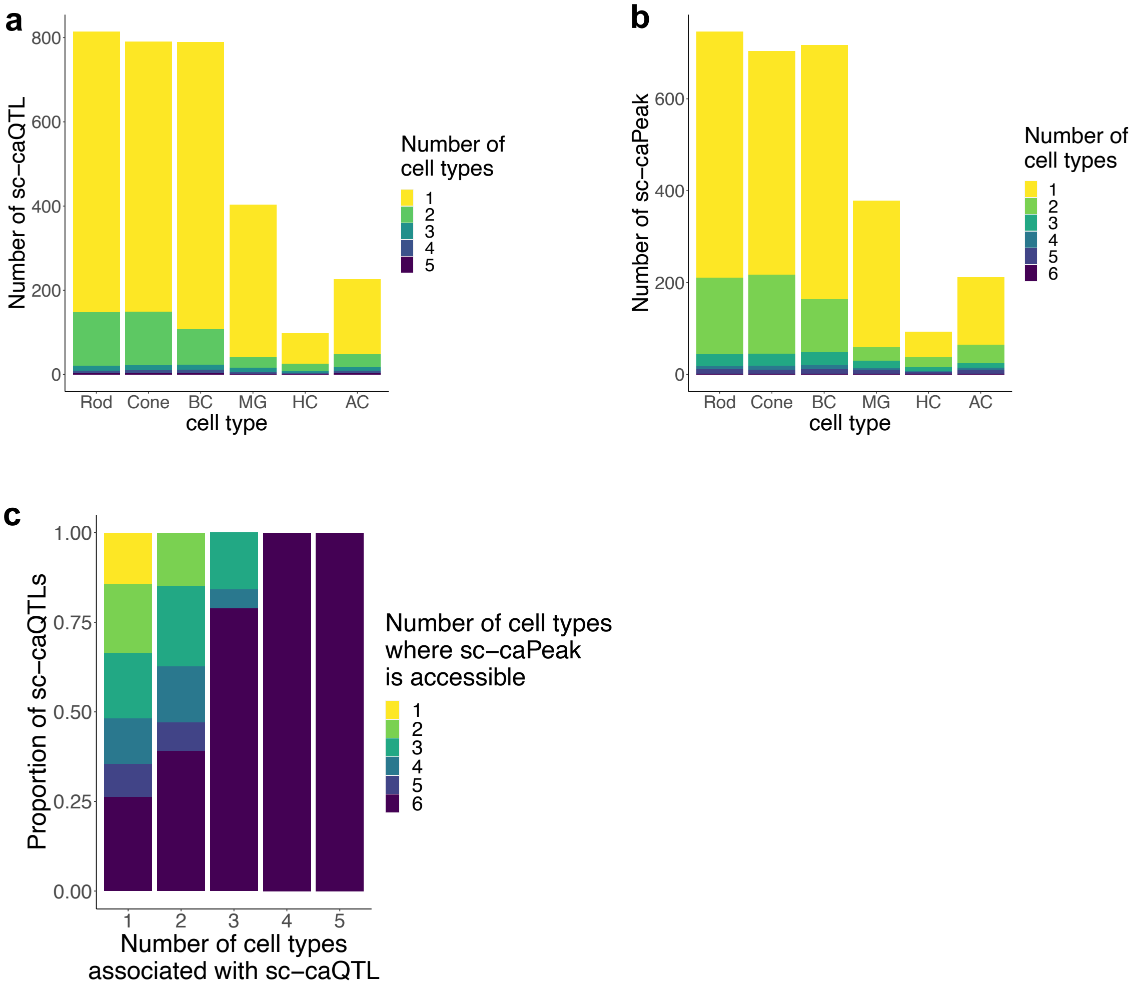

Fig. S4: Identification of sc-caQTL in down-sampled cells

a Bar plot showing the number of independent index sc-caQTLs reaching genome-level FDR < 0.1 per cell type, colored by the number of cell types where the sc-caQTL is significant. **b** Bar plot showing the number of sc-caPeaks reaching genome-level FDR < 0.1 per cell type, colored by the number of cell types where the sc-caPeak is significant. **c** Bar plot showing proportion of sc-caQTLs associated with sc-caQTLs that are significant in one or more cell types, colored by the number of cell types where the caPeak is accessible.

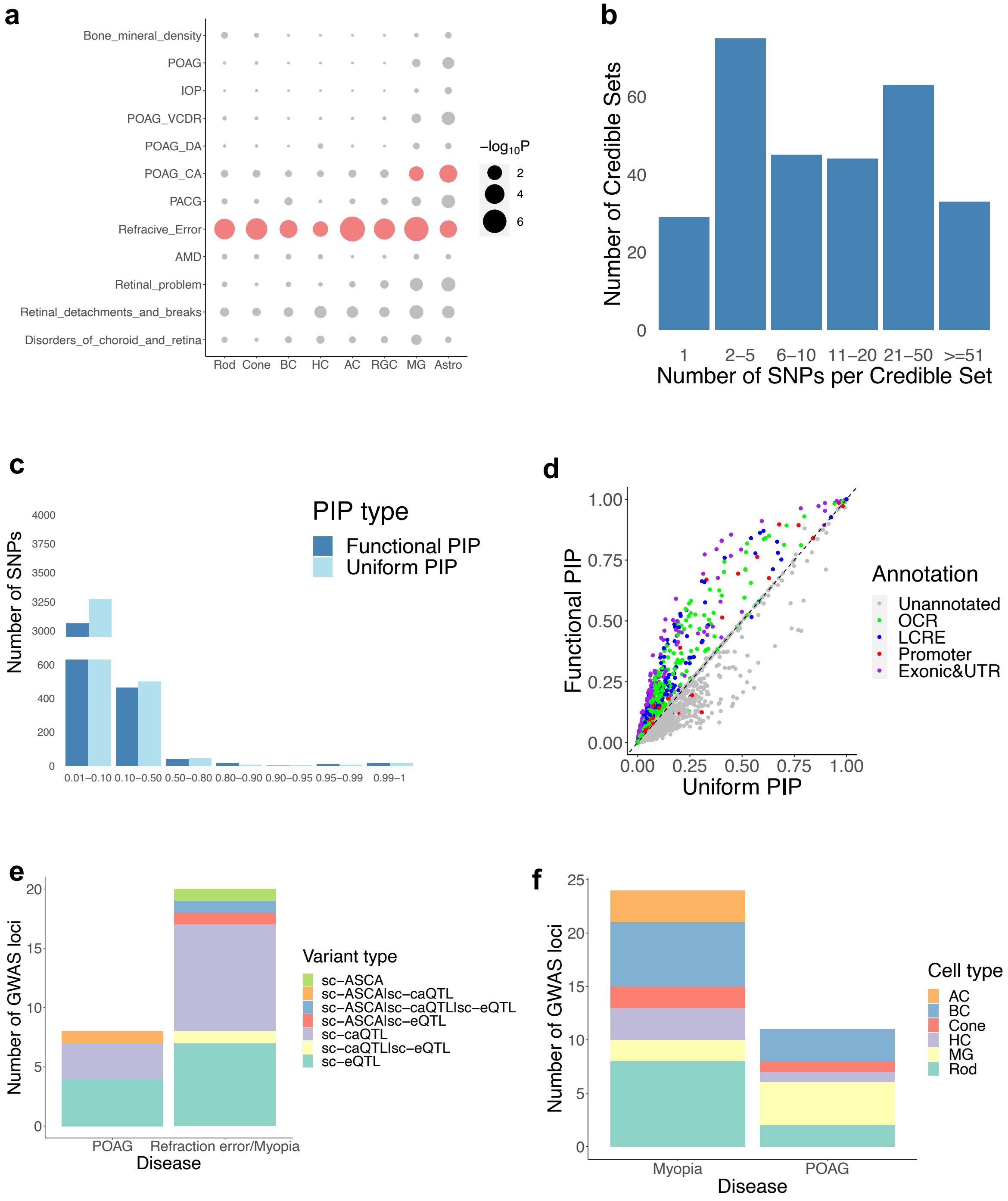

Fig. S5: Cell type enrichment and fine-mapping of GWAS loci.

a, Dot plot showing the enrichment of 11 eye-related and one control GWAS loci in the snATAC-peaks of retinal cell types, identified with LDSC. POAG: primary open-angle glaucoma. IOP: intraocular pressure. VCDR: vertical cup-disc ratio of optic nerve. CA: cup area of optic nerve. DA: disc area of optic nerve. PCAG: primary angle closure glaucoma. AMD: age-related

macular degeneration. The cell types enriched of GWAS traits with *q*-value < 0.1 are highlighted in red. **b,** Bar plot showing the distribution of SNP numbers per credible set. **c,** Bar plot showing the PIP value distribution of the fine-mapped SNPs. Functional PIP: PIP computed with functional annotation informed prior. Uniform PIP: PIP computed without functional annotation informed prior. **d,** Scatter plot showing the Uniform PIP (X axis) and Functional PIP (Y axis) of the GWAS variants. The variants in regulatory regions were prioritized with functional annotation informed prior (functional PIP), compared to the uniform PIP. n = 52087 SNPs. **e,** Bar plot showing the number of fine-mapped GWAS variants (Functional PIP > 0.1) overlapped with sc-QTL and/or ASCA for two GWAS traits. **f**, Bar plot showing the cell type distribution of fine-mapped GWAS variants (Functional PIP > 0.1) overlapped with sc-QTL/ASCA, colored by the cell type where the sc-QTL/ASCA is significant.

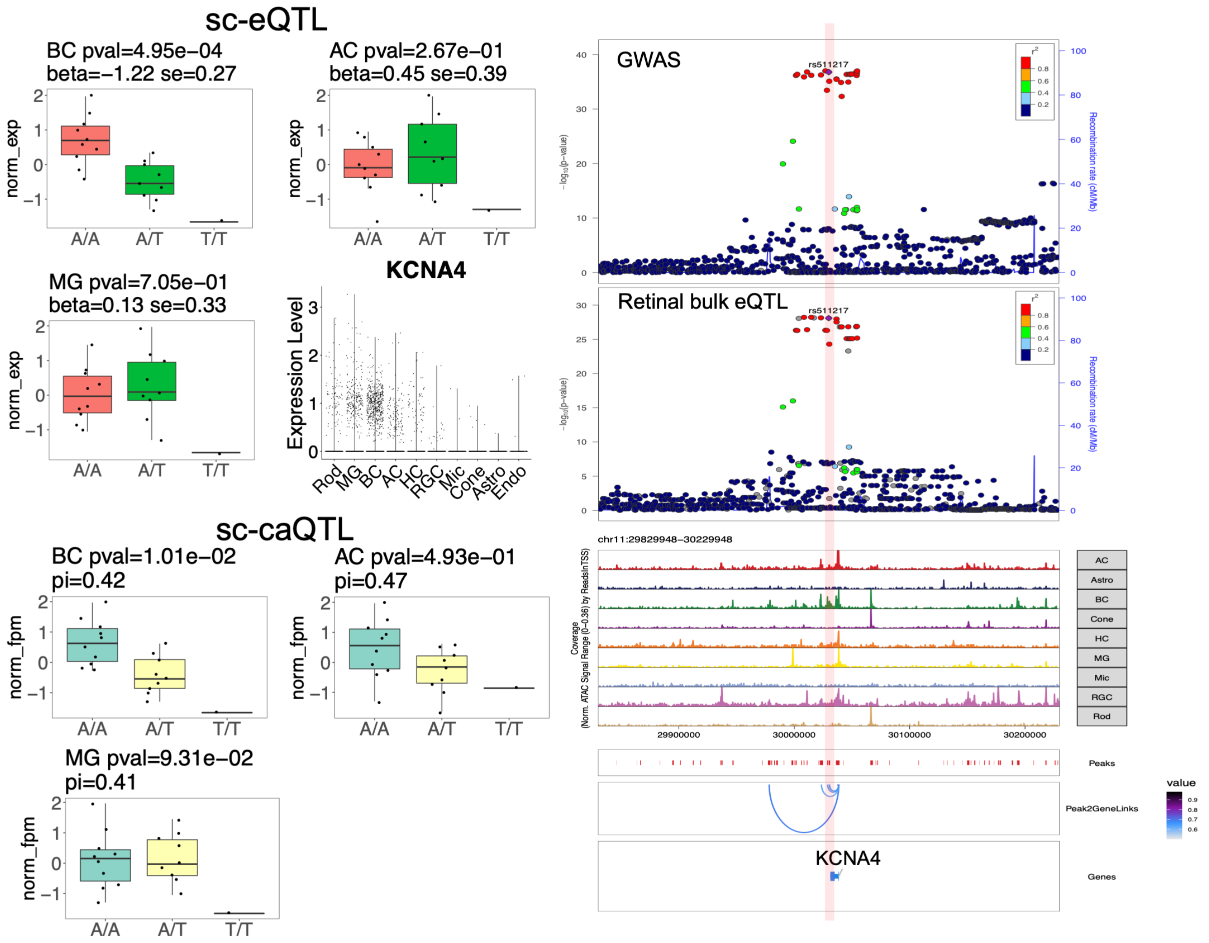

Fig. S6: An example of the fine-mapped GWAS candidate variants with the retinal bulk eQTL signal of its target gene colocalized with GWAS signal.

Box plot showing the gene expression level of *KCNA4* in 20 individuals with different genotype of rs511217 in BC, AC and MG (left top). Box plot showing the chromatin accessibility of the OCR harboring rs511217 in 20 individuals with different genotype of rs511217 in BC, AC and MG (left bottom). locusZoom plots showing the refraction error and myopia GWAS signal and bulk retinal eQTL signal ($-log_{10}(p - value)$) of rs511217 and the surrounding SNPs. Genome track of *KCNA4* locus showing cell type specific chromatin accessibility and positioning of rs511217 within an OCR, which is a predicted LCRE of *KCNA4*.

Supplementary Note

The selection of covariates for eQTL mapping

Given our relatively small cohort of N=20, we made a careful decision to include N=3 PEER factors and 1 genotype principal component (PC) as covariates. This choice was made to ensure sufficient statistical power, prevent overfitting, account for the systematic confounding effects in gene expression with the PEER factors, and enable accurate estimation of each variable in the linear regression (specifically, each variable in the linear regression can be estimated with 20/5=4 subjects).

The observed correlations between the top PEER factors and the quality control metrics of snRNA-seq suggested that these PEER factors correspond to known technical covariates. Specifically, in the case of rod cells, the 1st PEER factor is positively correlated with percent of reads mapped antisense to gene (Pearson correlation r= 0.51, p=0.02); the 2nd PEER factor is positively correlated to median UMI counts per cell (r= 0.47, p=0.04); and the 3rd PEER factor is positively correlated to fraction of reads in cells (r=0.77, p= 7.14e-05).

To assess the impact of including additional PEER factors in eQTL analysis, we conducted eQTL analysis using N=7 PEER factors and 1 genotype principal component (PC). Surprisingly, we observed a significant reduction in detection power, as the number of gene-level significant eQTLs decreased from 14,377 to 3,714. This finding suggests that including a higher number of PEER factors for a small sample cohort might introduce excessive correction and inadvertently suppress true eQTL signals. Furthermore, we performed another eQTL analysis incorporating 3 PEER factors, 1 genotype PC, age, and sex as covariates. Similarly, this analysis also resulted in a reduced power to detect eQTLs, with the number of gene-level significant eQTLs decreasing from 14,377 to 12,567. Notably, we observed a negative correlation between the 3rd PEER factor and the age of the samples (Pearson correlation r=-0.50, p=0.02). This finding suggests that the 3rd PEER factor partially captures the age effect in the data.

Considering these results, we have made the decision to utilize the current set of covariates, which includes 3 PEER factors and 1 genotype PC. This choice balances the need for confounding correction while preserving the detection power of eQTL analysis.

Assessing the potential bias in caQTL detection introduced by cell type abundance differences

To assess the impact of cell type abundance on sequencing depth (in cell type level) and thereby the number of sc-caQTLs detected per cell type, we performed a random down-sampling approach. We randomly sampled 400 cells per cell type per individual from the original dataset, allowing for sampling with replacement. Through this analysis, we consistently observed trends that aligned with our previous findings based on the original dataset. A total of 2,840 sc-caQTLs were detected, ranging from 98 to 814 per cell type. Notably, the majority of these sc-caQTLs were cell type-specific, with 74.5% to 90.1% being unique to a single cell type (Additional file1: Fig. S4a, b). Furthermore, we still found that the majority (85.7%) of the cell type specific sc-caQTLs were associated with caPeaks (namely, the residing peaks of caQTLs) that are accessible across multiple cell types (Additional file1: Fig. S4c). Overall, by conducting this random sampling analysis, we have addressed the potential bias introduced by cell type abundance differences, and our results consistently validated the patterns observed in the original dataset.

A comparison of fine-mapping using single-cell multi-omics data with using existing data from bulk eQTL and annotated cis-regulatory regions

Among a total of 9762 SNPs tested for GWAS, 1130 SNPs were also tested for single cell QTLs/ASCAs in our study, and 224 (19.8%) of the 1130 SNPs were overlapped with QTLs/ASCAs. In contrast, among a total of 816 fine-mapped GWAS, 261 SNPs were tested for single cell QTLs/ASCAs in our study, and 38 (14.6%) of the 261 SNPs were overlapped with single cell QTLs/ASCAs. Therefore, we did not observe a higher fraction of the fine-mapped GWAS SNPs overlapped with QTLs/ASCAs (14.6%) than expected (19.8%) (two-sided Fisher's Exact test, p=0.053). The potential reasons may be: 1) the small cohort of N=20 may result in reduced power for detecting QTL. 2) The examined GWAS loci might have higher overlapping rate with the QTLs from other tissue or cell types outside of retina. Specifically, age-related degeneration GWAS loci may be enriched in retinal pigment epithelium, myopia/fraction error and open-angle glaucoma loci may be also enriched in the anterior segment of the eye.

To evaluate if single-cell multiomics analyses enhance the power to nominate potential causal variants for GWAS traits, we compared the above findings with results from bulk sequencing data and existing data [85]. Specially, we performed the fine-mapping of GWAS variants using the annotated cis-regulatory regions from the ENCODE cCRE registry (N= 959,449 regions) 14 and identified 841 GWAS variants with PIP > 0.1, which is comparable to the number based on our single-cell multiomics study (N=816). Out of the 841 fine-mapped GWAS variants, 826 were tested for bulk retinal eQTLs. Among the tested variants, 216 (26.15%) overlap with bulk retinal eQTLs, showing 1.79-fold higher mapping rate compared to the single cell QTLs/ASCAs (two-sided Fisher's Exact test, $p = 7.7 \times 10^{-5}$). Considering that the bulk retinal eQTL study was conducted with a cohort of 22.65-fold more individuals (N=453) compared to our single cell association study (N=20), our single-cell multiomics and single-cell QTL mapping methods exhibit increased power in nominating potential causal variants for these GWAS variants. Furthermore, since gene regulation is highly cell type/context dependent, our single cell study provides a higher resolution in identifying the relevant cell types where these potential causal variants exert their effects.

Acronyms list

sc-eQTL: single-cell expression quantitative trait loci
sc-caQTL: single-cell chromatin accessibility quantitative trait loci
sc-ASCA: single-cell allelic specific chromatin accessibility
sc-ASE: single-cell allelic specific expression
GWAS: genome wide association study
WGS: whole genome sequencing
snRNA-seq: single nuclei RNA-sequencing
snATAC-seq: transposase-accessible chromatin sequencing
QC: quality control
Rod: rod photoreceptor cells
Cone: cone photoreceptor cells
BC: Bipolar cells
AC: amacrine cells
HC: horizontal cells
MG: müller glia cells

RGC: retinal ganglion cells
Astro: astrocytes
Endo: endothelial cells
Mic: microglia cells
OCRs: open chromatin regions
TF: transcription factor
CRE: cis regulatory elements
LCREs: linked cis regulatory elements, namely the cis regulatory elements that are assigned to target gene.
DARs: differentially accessible regions
TSS: transcription start site
FDR: false discovery rate
LD: linkage disequilibrium
eGene: the target gene of eQTL
caPeaks: the target peak of caQTL
MPRA: Massive Parallel Reporter Assay
Oligos: oligonucleotides
ChIP-seq: Chromatin immunoprecipitation followed by sequencing
POAG: primary open-angle glaucoma
CA: cup areas of optic nerve
VCDR: vertical cup-disc ratio of optic nerve
IOP: intraocular pressure
PIP: posterior inclusion probability
MDS analysis: multidimensional scaling analysis

Gene names:
OTX2, CRX, MEF2D, ONECUT2, NFIA, NFIB, NFIX, LHX2
SLC27A6, FOXP2, NR3C1, REST, NFE2L2, GRIN2A, EPAS1, WWC1, PEAK1
NRL, CTCF, KCNA4, GPC6
